# Distinct Roles of NANOS1 and NANOS3 in the Cell Cycle and NANOS3-PUM1-FOXM1 Axis to Control G2/M Phase in a Human Primordial Germ Cell Model

**DOI:** 10.3390/ijms23126592

**Published:** 2022-06-13

**Authors:** Erkut Ilaslan, Krystyna Kwiatkowska, Maciej Jerzy Smialek, Marcin Piotr Sajek, Zaneta Lemanska, Matisa Alla, Damian Mikolaj Janecki, Jadwiga Jaruzelska, Kamila Kusz-Zamelczyk

**Affiliations:** 1 Institute of Human Genetics, Polish Academy of Sciences, 60-479 Poznan, Poland; krystyna.kwiatkowska@igcz.poznan.pl (K.K.); maciej.smialek@igcz.poznan.pl (M.J.S.); marcin.sajek@igcz.poznan.pl (M.P.S.); zaneta.lemanska@igcz.poznan.pl (Z.L.); matisa.alla@igcz.poznan.pl (M.A.); djanecki@ibch.poznan.pl (D.M.J.); jadwiga.jaruzelska@igcz.poznan.pl (J.J.); 2 Biozentrum, University of Basel, 4056 Basel, Switzerland; 3 RNA Bioscience Initiative, University of Colorado School of Medicine, Aurora, CO 80045, USA; 4 Department of Biochemistry and Molecular Genetics, University of Colorado School of Medicine, Aurora, CO 80045, USA

**Keywords:** NANOS1, NANOS3, PUM1, FOXM1, human primordial germ cells, cell cycle, germ cell cancer

## Abstract

Nanos RNA-binding proteins are critical factors of germline development throughout the animal kingdom and their dysfunction causes infertility. During evolution, mammalian Nanos paralogues adopted divergent roles in germ cell biology. However, the molecular basis behind this divergence, such as their target mRNAs, remains poorly understood. Our RNA-sequencing analysis in a human primordial germ cell model-TCam-2 cell line revealed distinct pools of genes involved in the cell cycle process downregulated upon NANOS1 and NANOS3 overexpression. We show that NANOS1 and NANOS3 proteins influence different stages of the cell cycle. Namely, NANOS1 is involved in the G1/S and NANOS3 in the G2/M phase transition. Many of their cell cycle targets are known infertility and cancer-germ cell genes. Moreover, NANOS3 in complex with RNA-binding protein PUM1 causes 3′UTR-mediated repression of *FOXM1* mRNA encoding a transcription factor crucial for G2/M phase transition. Interestingly, while NANOS3 and PUM1 act as post-transcriptional repressors of FOXM1, FOXM1 potentially acts as a transcriptional activator of NANOS3, PUM1, and itself. Finally, by utilizing publicly available RNA-sequencing datasets, we show that the balance between FOXM1-NANOS3 and FOXM1-PUM1 expression levels is disrupted in testis cancer, suggesting a potential role in this disease.

## 1. Introduction

Post-transcriptional regulation of gene expression (PTGR) is a crucial regulatory step enabling the precise control of gene expression rate, timing, and localization within the cell. PTGR plays an essential role in germ cell development [1]. Thus, post-transcriptional regulators such as RNA-binding proteins (RBPs) are directly linked to disorders, including infertility and germ cell cancers [2,3,4]. Nanos is an evolutionary conserved RBP, identified as *Drosophila* morphogen crucial for abdomen patterning [5] and multiple aspects of germline biology, such as germ cells specification [6], their migration to primary gonads [7,8], regulation of germ cell apoptosis [9], and maintenance of germline stem cells [10]. During evolution, with the emergence of three Nanos paralogues (Nanos1, Nanos2, and Nanos3) in mammals, each of them acquired different expression patterns and distinct roles in germline development [11,12,13]. In humans, expression of NANOS3 is initiated at the 11th day of embryonic development at the time of human primordial germ cell (hPGC) specification and it is considered as a marker of hPGCs [14]. Simultaneously, NANOS1 expression is stimulated by the PRDM14 transcription factor, which is critical for hPGCs specification [15]. Both NANOS1 and NANOS3 are also expressed in hPGCs after they colonize the gonads, in male as well as female embryos [16]. In turn NANOS2 expression is not observed either in premigratory or in early post migratory hPGCs [16]. It starts much later than its paralogues, specifically in gonocytes of human male embryos and is male sex restricted [17]. In adult human gonads, expression of NANOS1 and NANOS2 are restricted to the male germline, ranging from spermatogonia to round spermatids [12,17], while NANOS3 is expressed in both sexes, in spermatogonia cells and follicle cells [18]. The importance of *NANOS1* and *NANOS3* for human fertility was demonstrated by identification of mutations in those genes in infertile patients. Namely, a mutation of *NANOS1* is associated with male infertility caused by absence of germ cells in the seminiferous tubules of patients [19]. Moreover, when this *NANOS1* gene mutation was tested in a hPGCs model, TCam-2 cell line, it resulted in a functional switch of NANOS1 from anti-apoptotic to pro-apoptotic, providing an explanation of the infertility phenotype of the patients [20]. In turn, *NANOS3* gene mutations were found to be associated with premature ovarian insufficiency, a female infertility phenotype [21,22]. Although no mutations related to male infertility have been found in this gene so far [23], a knockout of murine *Nanos3* resulted in infertility of both sexes [13], which is in accordance with similar murine and human expression patterns of those orthologues.

In an in vitro system, it was shown that upon binding to target mRNAs, Nanos homologues recruit a CCR4-NOT complex for deadenylation and promote repression by shortening the poly(A) tail. This recruitment is mediated through the N-terminal region which contains the conserved NIM domain (NOT1 Interacting Motif) acting as a binding site for CCR4-NOT deadenylase complex [24]. Besides the NIM domain, Nanos proteins contain a conserved (CCHC)2 type zinc finger domain, which potentially mediates target mRNA binding either by direct interaction [25] or indirectly by serving as a binding site for sequence specific RBPs such as a human Pumilio homologue, PUM2 [12]. Human Pumilio paralogues (PUM1 and PUM2) are members of the PUF family and contain the PUF RNA binding domain, which recognize the UGUAHAUA motif (commonly referred to as PUM binding element-PBE), located mostly in 3′UTR of target mRNAs [26].

Since Pumilio and not Nanos is responsible for specific nucleotide motif recognition in the 3′UTRs, studies on the mechanisms of post-transcriptional regulation were primarily focused on Pumilio while much less on Nanos. Moreover, unlike Pumilio proteins, a global identification of RNA targets of NANOS1 and NANOS3 proteins has not been reported so far. Although knockout mice models of Pumilio proteins demonstrated some effects on fertility [27,28], the underlying subfertility phenotypes are milder compared to Nanos knockout models, which result in complete infertility. This highlights the crucial roles of Nanos proteins in germ cell biology which is not a mere result of Nanos proteins acting as a cofactor of Pumilio proteins. Thus, in this study we sought to elucidate the molecular basis underlying the role of two human NANOS paralogues, NANOS1 and NANOS3, in human germ cell development.

Here we report the first global transcriptomic analysis of NANOS1 and NANOS3 mRNA targets, performed in a hPGC model, TCam-2 cell line. This cell line is the only existing human germ cell model in culture and represents an early postmigratory stage of hPGCs development. We show that many genes related to germ cell development, fertility, as well as cancer are downregulated upon Nanos overexpression. Furthermore, many of these germ cell development genes are involved in the cell cycle and NANOS1 as well as NANOS3 have a distinct effect on this process. Finally, we characterize FOXM1 as a target of NANOS3/PUM1 post-transcriptional repressor complex and elucidate the role of NANOS3-PUM1-FOXM1 axis in regulating G2/M phase cell cycle mRNAs, as well as its dysregulation in testis cancers.

## 2. Results

### 2.1. NANOS1 and NANOS3 Overexpression Cause Downregulation of Infertility and Cancer-Germ Cell Genes

**Figure 1:**
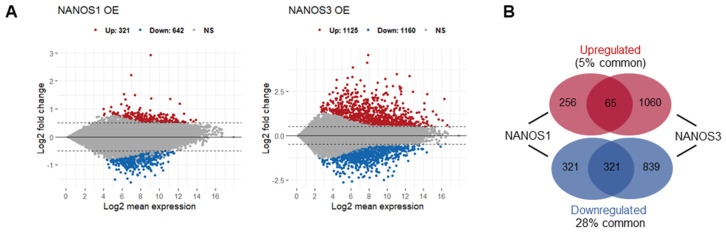

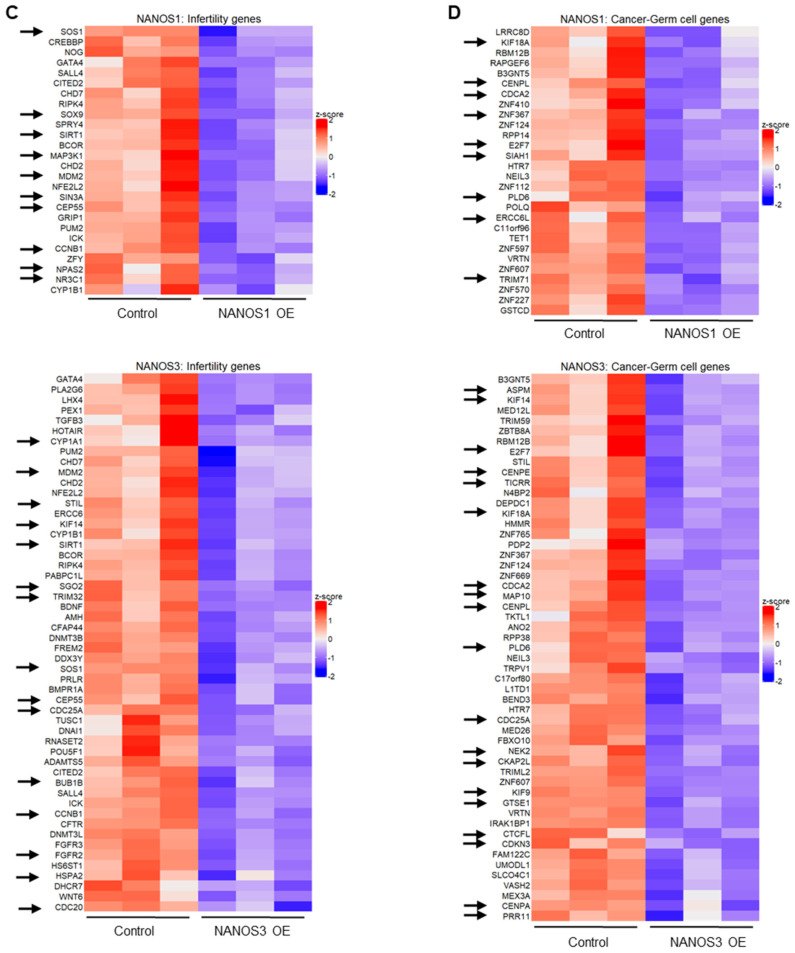
Transcriptomic changes upon NANOS1 and NANOS3 overexpression in TCam-2 cell line. (**A**) RNA-Sequencing (RNA-Seq) upon NANOS1 (left panel) and NANOS3 (right panel) overexpression visualized as MA-plots. Differentially expressed genes were filtered with log2FC ≥ 0.5 for upregulated and ≤ −0.5 for downregulated genes. Adjusted *p*-value ≤ 0.01 was considered as significant. (**B**) Venn diagram showing common and distinct differentially expressed genes between NANOS1 and NANOS3 overexpression. (**C**) Expression level of infertility genes downregulated upon NANOS1 (upper heatmap) and NANOS3 (lower heatmap) overexpression visualized as heatmaps with z-score scaling. (**D**) Cancer-germ cell genes downregulated upon NANOS1 (upper heatmap) and NANOS3 (lower heatmap) overexpression. Genes involved in cell cycle are marked by arrows.

In order to elucidate the role of NANOS1 and NANOS3 proteins in hPGCs development, we aimed to identify mRNAs potentially regulated by these proteins in the TCam-2 cell line. We did not include NANOS2 in this study because it is not expressed at that early stage of germline development represented by the TCam-2 cells [16]. Our transcriptome analysis of this cell line by RNA-sequencing (RNA-Seq) showed that both NANOS1 and NANOS3 are expressed at low levels at that stage (1.7 and 2.1 FPKM, respectively) [29]. Therefore, we overexpressed NANOS1 and NANOS3, followed by RNA sequencing (RNA-Seq) and differential gene expression (DGE) analysis to identify mRNAs potentially downregulated by these post-transcriptional repressors. Upon NANOS1 and NANOS3 overexpression, we identified that 642 and 1160 mRNAs were downregulated while 321 and 1125 mRNAs were upregulated, respectively (Figure 1A, Table S1). Among them, 65 upregulated and 321 downregulated mRNAs were identified as common for both NANOS1 and NANOS3 (Figure 1B). Gene ontology (GO) analysis of these differentially expressed genes showed enrichment of downregulated genes involved in differentiation and development processes including reproductive system development which contains genes such as *PRDM1* and *TFAP2C*, important factors for germ cell development.

In order to identify NANOS downregulated genes associated with infertility and/or germ cell cancers, we have curated a human infertility database by combining the existing data (MalaCards.org [30], KEGG Disease [31], eDGAR [32], NCBI-Gene [33], NCBI-GTR [22,33,34,35] (Table S3) and we have used a set of genes that are enriched in human male germ cells and cause cancer when abnormally expressed [36] (Table S4). We refer to the latter group of genes as cancer-germ cell genes. We identified 26 and 52 infertility genes downregulated (Figure 1C, Table S3) as well as 28 and 53 cancer-germ cell genes downregulated (Figure 1D, Table S4) for NANOS1 and NANOS3, respectively. Interestingly, many of these genes are involved in the cell cycle, i.e., 10/26 (34%) NANOS1 and 15/52 (27%) NANOS3 infertility genes as well as 9/28 (32%) NANOS1 and 19/53 (36%) NANOS3 cancer-germ cell genes are cell cycle related (Figure S2). Such enrichment was not observed in case of upregulated genes (Figure S1A,B)

Taken together, we show hundreds of genes differentially expressed in the TCam-2 cell line upon overexpression of NANOS1 and NANOS3. Many of these genes known to be important for human germ cell biology are also involved in the cell cycle.

### 2.2. Biological Clustering Analysis of NANOS1 and NANOS3 Downregulated Genes Reveals Cell Cycle Related Clusters Containing Infertility and Cancer-Germ Cell Genes

**Figure 2:**
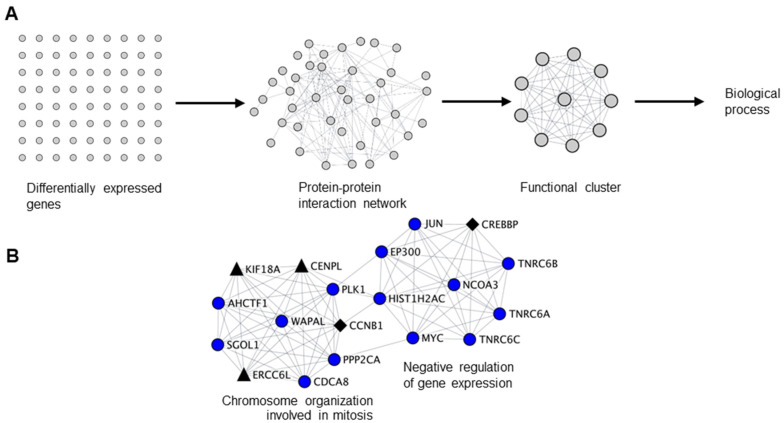

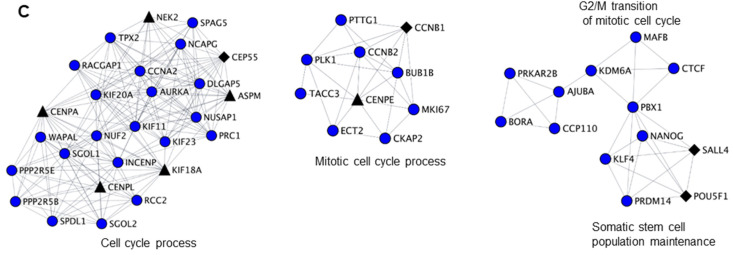
Biological clustering analysis (BCA) reveals cell cycle related functions for NANOS1 and NANOS3. (**A**) Workflow for biological clustering analysis (BCA). High confidence (score ≥ 0.9) protein–protein interaction networks of differentially expressed genes are created using the STRING database followed by cluster detection by MCODE. Further, these clusters were subjected to gene ontology (GO) search for the corresponding biological process. (**B**) NANOS1 and (**C**) NANOS3 downregulated cell cycle cluster containing infertility (shown in black diamonds, Supplementary Table S3) and cancer-germ cell genes (shown in black triangles, Supplementary Table S4).

Since we have identified cell cycle related functions as a common theme for both infertility and cancer-germ cell genes downregulated by NANOS1 and NANOS3, we sought to interrogate the functional relationship between them. For that purpose, we have designed a network biology approach that we refer to as biological clustering analysis (BCA). Briefly, in BCA we generated high confidence protein-protein interaction networks based on the differential gene expression data utilizing the interaction data from STRING database [37] and further selected functional clusters from the protein-protein interaction networks by utilizing the topological features using MCODE algorithm [38]. Finally, we performed GO analysis on identified clusters in order to reveal their function (Figure 2A). BCA of RNA-Seq results obtained upon NANOS1 and NANOS3 overexpression revealed 9 functional clusters containing downregulated genes in the case of NANOS1 and 20 functional clusters containing downregulated genes in the case of NANOS3 (Table S5). As the next step we screened these clusters to check whether they contain infertility or cancer-germ cell genes. This search revealed that in the case of NANOS1 overexpression, three infertility related genes (*CREBBP*, *CCNB1*, and *NPAS2*) and five cancer-germ cell genes (*CENPL*, *ERCC6L*, *KIF18A*, *SIAH1*, and *TRIM71*) were present in three clusters (Figure 2B and Figure S3). In the case of NANOS3 overexpression, 14 infertility related genes (*BUB1B*, *CCNB1*, *SALL4*, *POU5F1*, *TRIM32*, *CDC20*, *CFTR*, *TGFB3*, *DHCR7*, *SIRT1*, *HSPA2*, *ADAMTS5*, *BMR1A*, and *WNT6*) and 10 cancer-germ cell genes (*NEK2*, *CENPA*, *CENPL*, *KIF18A*, *ASPM*, *CENPE*, *FBXO10*, *KIF9*, *MED26*, and *SLCO4C1*) were present in 8 clusters (Figure 2C and Figure S4). Finally, many of these infertility and cancer-germ cell genes are present together in cell cycle related clusters downregulated by NANOS1 (Figure 2B) and NANOS3 (Figure 2C), suggesting a potential role of NANOS-mediated regulation of cell cycle process for proper human germ cell development and fertility.

### 2.3. Distinct Phenotypic Effects of NANOS1 and NANOS3 on Cell Cycle

**Figure 3:**
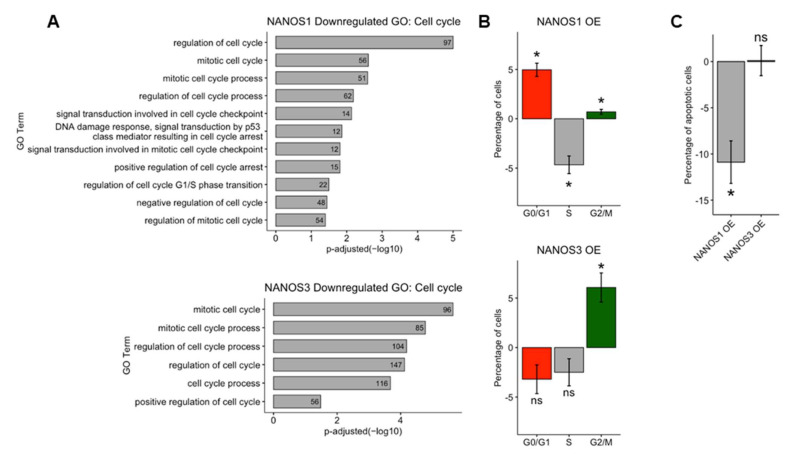
Distinct phenotypic effects of NANOS1 and NANOS3 overexpression on the cell cycle. (**A**) Cell cycle related biological processes downregulated upon NANOS1 (top panel) and NANOS3 (bottom panel) overexpression identified in GO analysis. (**B**) Cell cycle analysis by propidium iodide staining followed by flow cytometry analysis upon NANOS1 (top panel) and NANOS3 (bottom panel) overexpression. (**C**) Apoptosis analysis by Annexin-V staining followed by flow cytometry analysis upon NANOS1 and NANOS3 overexpression. Standard error is visualized as error bars. * = *p*-value ≤ 0.05.

The results of RNA-Seq, BCA, and GO analysis of NANOS downregulated genes indicate that both NANOS1 and NANOS3 proteins play a role in regulation of the cell cycle in TCam-2 cells. To further explore the effect of these proteins on the cell cycle, we overexpressed NANOS1 and NANOS3 and performed cell cycle analysis by propidium iodide staining followed by flow-cytometry analysis. In the case of NANOS1 overexpression, we observed a higher number of cells in G1 and G2/M phases and fewer in S phase compared to control cells, (Figure 3B top panel). The effect of NANOS1 on the cell cycle is consistent with GO indication that NANOS1 represses genes involved in the G1/S phase transition (Figure 3A top panel, Table S2). Moreover, GO analysis indicates that NANOS1 represses genes involved in the p53 mediated DNA damage checkpoint process which takes place during S phase. Downregulation of this phase accelerates both cell proliferation and S phase [39]. These results are in line with our previous findings that NANOS1 downregulates GADD45 family members which are involved in G1/S phase DNA damage checkpoint [20]. In turn, in the case of NANOS3 overexpression, we observed a higher number of cells in the G2/M phase compared to control cells (Figure 3B bottom panel). The effect of NANOS3 on the cell cycle is also consistent with GO indication that NANOS3 represses a high number (96) of mitotic cell cycle genes (Figure 3A bottom panel, Table S2).

As stated above, NANOS1 overexpression demonstrate anti-apoptotic properties through downregulation of pro-apoptotic cell cycle genes such as GADD45 family members. Since overexpression of NANOS1 and NANOS3 results in distinct cell cycle phenotypes, we asked whether the effect of NANOS3 overexpression also influences apoptosis of TCam-2 cells. For that purpose, we performed Annexin V staining followed by flow-cytometry analysis in order to detect apoptotic cell population. In contrast to NANOS1, overexpression of NANOS3 did not lead to a meaningful change in the apoptotic cell population (Figure 3C and Figure S5).

Taken together, we show the distinct effects of NANOS1 and NANOS3 proteins on the cell cycle and apoptosis in TCam-2 cells which are in line with the transcriptomic changes induced by overexpression of these proteins.

### 2.4. TAF1 and FOXM1 Are Transcriptional Regulators for NANOS1 and NANOS3 Downregulated Genes

Next, we asked whether NANOS-dependent massive downregulation of cell cycle genes could be partially caused by activity of NANOS targets encoding transcription factors (TFs) involved in cell cycle regulation. In order to decipher this, we aimed to identify TFs that regulate cell cycle genes downregulated by NANOS1 and NANOS3. For that purpose, we utilized the publicly available ChIP-Seq data to check for promoter region binding of all NANOS1 and NANOS3 downregulated genes using iRegulon [40]. This analysis revealed TAF1 (TATA-box binding protein associated factor 1) as the most prominent transcription factor for promoter binding for NANOS1 downregulated genes, and FOXM1 (Forkhead Box M1) for NANOS3 downregulated genes (Figure S6A, Table S6). Additionally, we have performed gene overlap analysis to supplement iRegulon. This analysis showed that TAF1 and FOXM1 ENCODE targets significantly overlap with NANOS1 and NANOS3 downregulated genes, respectively. These results were in line with iRegulon analysis showing that both TAF1 and FOXM1 targets determined in the ENCODE project significantly overlap with NANOS1 and NANOS3 downregulated genes respectively (Figure S7A, Table S10). TAF1 is a transcription factor known for its importance in G1/S phase transition in a p53 dependent manner [41,42] which is in line with the cell cycle phenotype we observed upon NANOS1 overexpression. On the other hand, FOXM1 induces the expression of over a hundred genes important for cell cycle and regulates all stages in a spatiotemporal manner [34,43,44,45]. Moreover, comparison of iRegulon targets identified for TAF1-NANOS1 and FOXM1-NANOS3 showed mostly distinct genes regulated by these pairs (Figure S6B).

Taken together, we show that transcriptional targets of TAF1 strongly overlap with NANOS1 downregulated genes and FOXM1 with NANOS3. Intriguingly, both NANOS1 and TAF1 has ubiquitous expression profile across human tissues. On the other hand, NANOS3 and FOXM1 expression is enriched in human germ cells [18] and a significant fraction of NANOS3 downregulated infertility (19/52) and cancer-germ cell genes (28/53) are also FOXM1 transcriptional targets (iRegulon). This indicates a potential germ cell specific regulation of G2/M phase by NANOS3 and FOXM1. Thus, we further focused on elucidating the role of NANOS3 and FOXM1 in regulating G2/M phase.

### 2.5. NANOS3 and PUM1 Cooperate in Post-Transcriptional Repression of FOXM1-Regulated Genes

**Figure 4:**
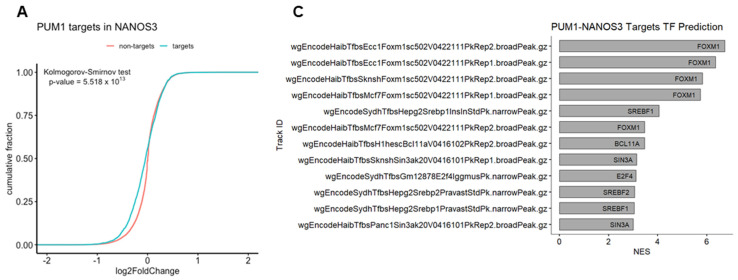

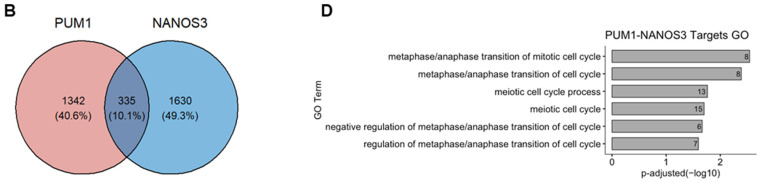
FOXM1 and TAF1 act as transcription factors for NANOS1 and NANOS3 downregulated genes. (**A**) Cumulative distribution analysis of differentially expressed genes upon NANOS3 RNA-Seq. Common differentially expressed mRNA distribution shown in blue and labeled as targets. The rest of the differentially expressed genes are shown in red and labeled as non-targets. (**B**) Common differentially expressed genes that are repressed by both NANOS3 and PUM1 identified as downregulated (log2FC < 0) upon NANOS3 overexpression and upregulated (log2FC > 0) upon PUM1 knockdown. (**C**) iRegulon transcription factor prediction analysis for NANOS3/PUM1 common repressed genes. (**D**) Cell cycle related biological processes identified by gene ontology enrichment analysis for NANOS3-PUM1 common repressed genes.

We have previously identified *FOXM1* mRNA as a direct target of PUM1 [29]. Thus, we asked whether NANOS3 and PUM1 cooperate in *FOXM1* mRNA repression. For that purpose, we have re-analyzed the PUM1 knockdown RNA-Seq data from TCam-2 cells with the same differential gene expression pipeline which was used in this study (Figure S6C). Using the empirical cumulative distribution function, we found significantly stronger downregulation of PUM1 targets vs. non-targets upon NANOS3 overexpression shown by a shift towards the left side in the cumulative distribution (Figure 4A). Additionally, comparison of NANOS3 downregulated genes with PUM1 eCLIP targets in the K562 cell line showed their significant overlap (Figure S7B, Table S10). Furthermore, we screened 3′UTR sequences of NANOS3 downregulated genes and performed motif enrichment analysis. This analysis revealed motifs corresponding to PBE in top 10 hits for NANOS3 downregulated genes. Such enrichment was not observed for NANOS3 upregulated genes (Table S11). Taken together, these results support NANOS3 and PUM1 cooperation. Therefore, we screened for common targets repressed by PUM1 and NANOS3. For this selection we filtered the genes by adjusted *p*-values only and included all the genes that are significantly downregulated upon NANOS3 overexpression and upregulated upon PUM1 knockdown, regardless of their fold change levels. We have identified 335 common genes for NANOS3 and PUM1 (Figure 4B). iRegulon analysis on these 335 genes showed FOXM1 as the most prominent transcription factor with binding to the promoter of 53 of them (Figure 4C and Figure S6D, Table S6). Furthermore, GO analysis on those 335 potential NANOS3 and PUM1 common targets showed enrichment of G2/M phase related genes (Figure 4D) which is in line with the cell cycle phenotype we observed upon NANOS3 overexpression.

### 2.6. FOXM1 Is a Post-Transcriptional Target for NANOS3/PUM1 Complex

**Figure 5:**
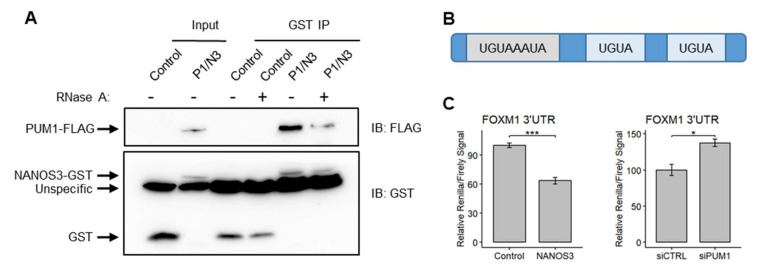
*FOXM1* mRNA is regulated by the NANOS3/PUM1 post-transcriptional complex through its’ 3′UTR. (**A**) Western-blot visualization of GST pull-down of NANOS3 with and without RNase A. (**B**) Schematic representation of *FOXM1* 3′UTR containing the Pumilio binding element (PBE-UGUAHAUA) and 2 PBE-like motifs (containing core UGUA motif). (**C**) Dual luciferase assay to measure Renilla/Firefly ratio upon PUM1 knockdown and NANOS3 overexpression using 3′UTR of *FOXM1* in the downstream of Renilla ORF. Standard error is visualized as error bars. * = *p*-value ≤ 0.05, *** = *p*-value ≤ 0.001.

Since NANOS3 and PUM1 potentially cooperate for mRNA repression in the TCam-2 cell line, we asked whether these two proteins also interact and form a protein complex. For this purpose, we performed pulldown experiments in which we overexpressed PUM1-FLAG and NANOS3-GST in the TCam-2 cell line in the presence and absence of RNAse A. This experiment showed that indeed NANOS3 and PUM1 can form a complex in the TCam-2 cell line. The lack of RNA weakened this interaction drastically (Figure 5A), suggesting that the presence of RNA potentially stabilizes NANOS3–PUM1 interaction. Next, we have investigated whether *FOXM1* mRNA is a NANOS3/PUM1 target. Since NANOS/PUM RNP complexes function by binding to the specific PBE motif (UGUAHAUA) in the 3′UTR of target mRNAs followed by repression, we screened the 3′UTR of *FOXM1* mRNA and identified one PBE and two PBE-like (containing UGUA core) motifs (Figure 5B). Thus, we hypothesized that *FOXM1* mRNA is regulated by NANOS3/PUM1 through its 3′UTR. To test this, we have cloned the 3′UTR of *FOXM1* downstream of the Renilla luciferase ORF and performed dual-luciferase experiments in the HEK293FT cell line. Upon overexpression of NANOS3 the Renilla/Firefly luciferase ratio significantly decreased; meanwhile, upon knockdown of PUM1, the Renilla/Firefly luciferase signal ratio increased (Figure 5C), indicating 3′UTR-dependent downregulation of *FOXM1* by NANOS3 and PUM1.

Taken together, we show that NANOS3 and PUM1 can bind to each other and form a complex in the TCam-2 cell line. Furthermore, we show that both NANOS3 and PUM1 are crucial for post-transcriptional repression of *FOXM1* via its 3′UTR.

### 2.7. NANOS3-PUM1-FOXM1 Axis Coordinates Expression of G2/M Phase Cell Cycle Genes

Among the genes we identified as potentially regulated by NANOS3, PUM1, and FOXM1, we have selected *CCNA2* encoding a cyclin which binds and activates CDK1 during G2 phase enabling G2/M phase transition [46], *KIF20B* encoding a kinesin protein highly phosphorylated and active during M stage [47], and *RAD21* encoding a DNA-binding protein involved in G2/M phase DNA damage checkpoint [48] as model genes involved in different aspects of G2/M phase to interrogate regulation of this process by NANOS3-PUM1-FOXM1 axis.

**Figure 6:**
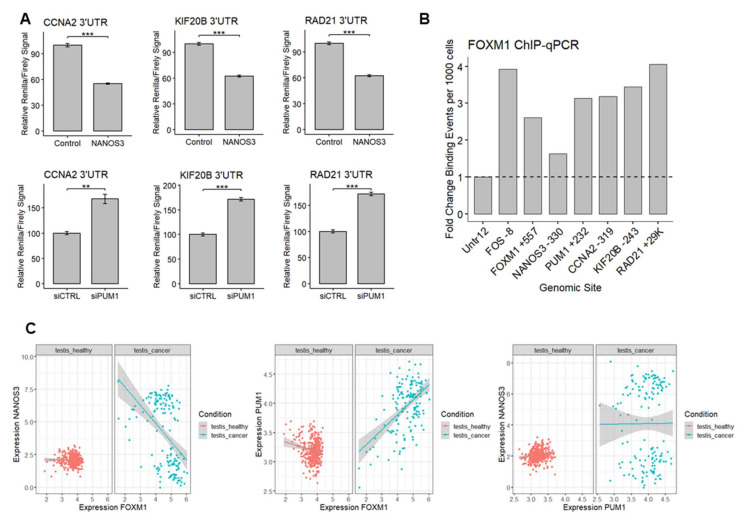
The NANOS3–PUM1–FOXM1 axis coordinates the expression of the cell cycle genes. (**A**) Dual luciferase assay to measure Renilla/Firefly ratio upon PUM1 knockdown and NANOS3 overexpression using 3′UTR of *CCNA2*, *KIF20B*, and *RAD21* in the downstream of Renilla ORF. (**B**) Relative fold change of FOXM1 binding events per 1000 cells for the promoter region of the target genes measured by ChIP-qPCR. (**C**) Differential correlation analysis of *NANOS3*, *PUM1*, and *FOXM1* in publicly available healthy testis (GTEx) and testicular cancer datasets (TCGA). Each dot represents a single GTEx or TCGA dataset. Expression values are transformed as log(expression +1). Standard error is visualized as error bars. ** = *p*-value ≤ 0.01, *** = *p*-value ≤ 0.001.

First, since *CCNA2*, *KIF20B*, and *RAD21* contain PBE elements in their 3′UTRs, we asked whether NANOS3 and PUM1 can regulate these mRNAs through their 3′UTRs. Thus, we performed additional dual luciferase experiments using 3′UTR of these genes in the downstream of Renilla luciferase ORF in HEK293FT cell line. Overexpression of NANOS3 led to decreased Renilla/Firefly luciferase signal ratio in all tested constructs (Figure 6A upper panel). Knockdown of PUM1 lead to increased Renilla/Firefly luciferase signal ratio in all of the tested constructs (Figure 6A lower panel), indicating 3′UTR mediated repression of *CCNA2*, *KIF20B*, and *RAD21* mRNAs by NANOS3 and PUM1.

Since potential FOXM1 target genes were selected from publicly available FOXM1 ChIP-Seq data from ENCODE representing other than the TCam-2 cell lines, we sought to validate FOXM1 binding to the promoter of *CCNA2*, *KIF20B*, and *RAD21* in TCam-2 cells using ChIP-qPCR. Additionally, our screening of ENCODE data showed that FOXM1 can potentially bind to the promoter regions of *NANOS3*, *PUM1*, and its own gene, therefore we also included these genes in the validation. The ChIP-qPCR experiment revealed enrichment of FOXM1 binding to all of the promoter regions that were tested (Figure 6B) indicating that *CCNA2*, *KIF20B*, *RAD21*, *NANOS3*, *PUM1*, and *FOXM1* genes are indeed transcriptional targets of FOXM1 in TCam-2 cells. In the case of *NANOS3*, FOXM1 binding was low, explaining at least in part the low expression level of *NANOS3* in TCam-2 cells.

Finally, we checked whether our findings on the functional relationship between NANOS3, PUM1, and FOXM1 in the TCam-2 cell line have an impact on human reproductive health. To this end we utilized the publicly available transcriptomic expression datasets from healthy and cancer testis samples and compared the correlation between *NANOS3*, *PUM1*, *FOXM1*, and the model cell cycle targets. This analysis showed a much wider expression pattern for *PUM1*, *NANOS3*, *FOXM1*, and target cell cycle genes in testis cancer compared to healthy testis samples (Figure 6C, Table S7) including altered NANOS3-FOXM1 and PUM1-FOXM1 correlations. This suggests dysregulation of the NANOS3–PUM1–FOXM1 axis in testis cancer compared to healthy testis. Taken together, we demonstrate the interplay between NANOS3, PUM1, and FOXM1 in regulating G2/M phase genes and disruption of this interplay in testicular cancer.

## 3. Discussion

Although mutations in *NANOS1* and *NANOS3* are associated with pathology of germ cells resulting in infertility, the underlying molecular mechanism of the function of NANOS proteins in the context of human germ cell development and biology remains elusive. Here we report the first global transcriptomic characterization of NANOS1 and NANOS3 target mRNAs in the TCam-2 cell line corresponding to the early post-migratory stage of hPGCs. We show that increasing the level of NANOS1 and NANOS3 proteins, which is an event occurring during the germ cell development, results in differential expression of hundreds of genes, involved mostly in the same developmental processes, including reproductive system development, differentiation, cell cycle, and transcriptional regulation. Interestingly, NANOS1 and NANOS3 proteins regulate these processes by distinct mRNA pools supporting the idea of NANOS paralogues acquiring distinct but complementary roles in germ cell development during evolution. It is important to highlight that we show the distinct influence on the cell cycle for each NANOS. Namely, upon NANOS1 overexpression, we observed more cells in G1 and G2/M phases and less cells in S phase while NANOS3 overexpression resulted in more cells in G2/M phase. However, future studies are required to establish whether NANOS1 and NANOS3 act in tandem thus at different stages of the cell cycle in germ cells or whether they exert their activity independently from each other at different time points during germ cell development.

Importantly, many of NANOS1 and NANOS3 target mRNAs represent genes previously identified as important factors for fertility as well as cancer-germ cell related genes. Our findings show that both infertility and cancer-germ cell NANOS target genes are enriched in cell cycle related functions. Dysregulation of cell cycle genes is a well-known cause of cancer [49] and many of the cancer-testis antigens carry out cell cycle related functions [50]. Thus, our findings support the notion of a link between infertility and cancer [51] through dysregulation of crucial cell cycle genes.

We show that FOXM1 may act as a transcription factor for many cell cycle genes that are downregulated by NANOS3 and PUM1. In mice, Foxm1 is a crucial factor for embryonal development and *Foxm1*^−/−^ mice embryos die in utero at the early stages of development. In these embryos, an increased number of polyploid cells were observed due to incomplete mitosis, suggesting a crucial role for FOXM1 in regulating the G2/M phase of the cell cycle during development (for review see [52]). Interestingly, following prenatal development, FOXM1 expression is dramatically reduced in human adult tissues, except in testis [18]. In testis, FOXM1 is highly expressed in spermatocytes, spermatids, and also in somatic Sertoli cells in seminiferous tubules [53,54]. This expression pattern suggests an important role of FOXM1 in spermatogenesis, yet the role of FOXM1 in germ cell biology remains unexplored. Our study proposes FOXM1 as a factor for the early stage of human germ cell development represented by the TCam-2 cell line. However, it is important to highlight that further studies in the emerging human germ cell models [14] is necessary to get a more complete picture of the role of FOXM1 in human germ cell development.

**Figure 7:**
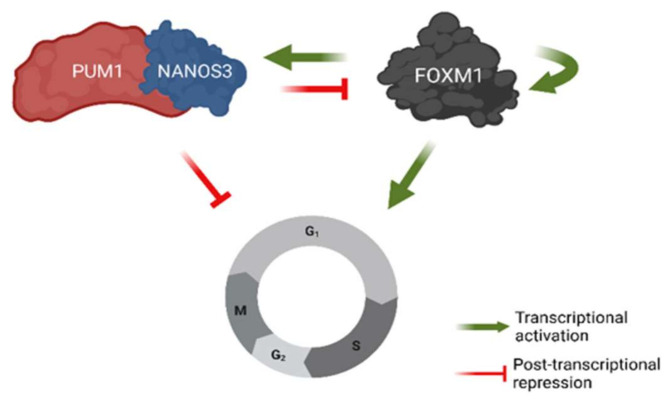
Model of NANOS3-PUM1-FOXM1 axis regulating G2/M phase of the cell cycle in TCam-2 cell line. Created with BioRender.com.

Our results suggest a mechanism, involving transcriptional and post-transcriptional feedback loops for the regulation of G2/M phase cell cycle genes (Figure 7). Namely, we show that NANOS3 and PUM1 form a complex in the TCam-2 cell line and act as post-transcriptional repressor of FOXM1. In return, FOXM1 acts as a potential transcriptional activator for both NANOS3 and PUM1. Furthermore, NANOS3, PUM1, and FOXM1 regulate common G2/M phase cell cycle genes demonstrating the role of FOXM1-NANOS3-PUM1 axis in controlling the G2/M phase. Our findings suggest that the NANOS3/PUM1 complex directly regulates at least part of the G2/M phase genes together with the indirect regulation via FOXM1. An interpretation of these results is that the FOXM1-NANOS3-PUM1 axis forms a feedforward-feedback loop [55], where PUM1 and NANOS3 buffer FOXM1 transcriptional noise for important cell cycle genes.

According to the differential correlation analysis of publicly available testis and testicular cancer expression datasets, the NANOS3–PUM1–FOXM1 axis is dysregulated in testis cancer. Previously it was shown that an abnormal expression of *NANOS* and *PUM* genes is observed in many types of cancer [56,57,58,59]. FOXM1 is a critical driver of proliferation and it is overexpressed in a broad range of cancers. Interestingly, its overexpression is most prominent in germ cell tumors [60]. A better understanding of FOXM1 function and regulation could lead to novel treatment strategies for a broad range of cancers, including germ cell tumors.

## 4. Materials and Methods

### 4.1. Cell Culture

TCam-2 cells were cultured in RPMI with GlutaMAX medium (Gibco #61870044) supplemented with 10% (*v*/*v*) fetal bovine serum (HyClone #sv 30160.03) and 1% (*v*/*v*) antibiotic/antimycotic solution (Lonza #17-602E). HEK293FT cells were cultured in low glucose DMEM medium (Lonza #BE12-707F) supplemented with 2 mM L-Glutamine (Thermo Fisher Scientific, Waltham, MA, USA, #25030081), 10% (*v*/*v*) fetal bovine serum (HyClone), and 1% (*v*/*v*) antibiotic antimycotic solution (Lonza).

### 4.2. Transfections

Mammalian cells were transfected with plasmids or siRNAs using the Neon Transfection System (Life Technologies), according to the manufacturer’s protocol, followed by culture in the same medium without antibiotic/antimycotic solution. Transient overexpression was performed by transfecting cells with pCMV6-entry vector (OriGene) containing *NANOS1* (#RC219123), *NANOS3* (#RC221867), and PUM1 (#RC201219) ORF tagged with FLAG (DYKDDDDK) peptide in the C-terminus. As negative control, cells were transfected with a pCMV6-entry vector containing only the sequence encoding FLAG peptide (referred as empty pCMV6-entry #PS100001). For pulldown experiments a pEBG vector containing *NANOS3* ORF tagged with GST in the C-terminus was used. As negative control, cells were transfected with a pEBG vector containing only the sequence encoding GST (Addgene #22227). For each transient overexpression 2 × 10^6^ cells were counted and transfected with 10 µg of respective plasmid. Cells were collected 24 h post transfection for downstream experiments. Transient knockdown by siRNAs was performed using silencer select siRNA against *PUM1* (Thermo Fisher Scientific, #s18681) and negative control siRNA (Thermo Fisher Scientific, #4390843). For each transfection 2 × 10^5^ cells were transfected with 80 pM siRNA and cells were collected 48 h post transfection for downstream experiments.

### 4.3. RNA-Sequencing

Total RNA was extracted using RNeasy Plus Micro Kit (Qiagen #74034) according to the manufacturer’s protocol. RNA quality was checked on Bioanalyzer (Agilent) using RNA 6000 Nano Kit (Agilent #5067-1511). Total RNA samples with RIN value > 7 were used for library preparation. cDNA libraries were prepared using TruSeq RNA Sample Prep v2 (Illumina #RS-122-2001) and subsequent next-generation sequencing was performed on an Illumina HiSeq 4000 platform by Macrogen INC. Sequencing from each sample was performed: 101nt long pair-end reads with at least 80 M reads per sample. Differential expression analysis was performed on the Galaxy platform by the following pipeline: Paired-End sequences obtained from the HiSeq 4000 platform were trimmed using the Trimmomatic v0.36.3 [61]. Sequence reads that pass quality filters were mapped to the human reference genome hg38 using Bowtie 2 v2.3.4.1 [62]. Mapped reads were counted using featureCounts v1.6.0.3 [63] followed by calculating differential gene expression with DESeq2 v2.11.40.1 [64]. Differentially expressed transcripts were filtered according to the parameters of log2FC ≥ 0.5 for upregulated and log2FC ≤ −0.5 for downregulated genes. Adjusted *p*-value ≤ 0.01 was considered as significant.

### 4.4. BCA (Biological Clustering Analysis)

BCA was performed in Cytoscape v3.6.1. Differentially expressed genes were filtered by their log2FC values (upregulated: ≥0.5, downregulated: ≤−0.5) and adjusted *p*-value ≤ 0.01. High confidence PPI networks were generated by using StringApp v1.6.0 [37] with the parameter confidence score ≥ 0.9. Functional clusters were generated by using MCODE v2.0.0 [38] with default parameters. Functions of the clusters were determined by using ClueGO v2.5.3 [65] with the GO/pathway selection parameter: minimum number of genes = (number of genes inside a given cluster-1). Gene ontologies with adjusted *p*-value ≤ 0.05 were considered as significant.

### 4.5. Gene Overlap Analysis of ENCODE Chip-Seq and eCLIP Data

Bed narrowPeak files for TFs chip-seq and RBPs eCLIP in K562 cells with hg38 annotation were downloaded from ENCODE. eCLIP files were first filtered using the following criteria: log2(eCLIP fold-enrichment over size-matched input) ≥ 3 and −log10(eCLIP vs. size-matched input *p*-value) ≥5. Peak annotation for both types of experiments was done using ChIPpeakAnno R library version 3.20.1 [66,67]. GRanges object was then transformed to a data frame using the annoGR2DF function from Repitools R library version 1.32.0 [68]. Overlapping between the datasets and NANOS1/NANOS3 differentially expressed genes was calculated using GeneOverlap R library version 1.22.0 [Shen L, Sinai ISoMaM (2019). GeneOverlap: Test and visualize gene overlaps. R package version 1.22.0, http://shenlab-sinai.github.io/shenlab-sinai/] (accessed on 21 February 2022). Overlaps with *p* value < 0.01 were assessed as significant ones. Top 15 hits with the highest odds ratio for every category (NANOS1 downregulated, NANOS1 upregulated, NANOS3 downregulated, and NANOS3 upregulated) were plotted as a heatmap using pheatmap R library version 1.0.12 (Raivo Kolde (2019). The pheatmap was performed using Pretty Heatmaps. R package version 1.0.12. https://CRAN.R-project.org/package=pheatmap) (accessed on 21 February 2022) without rows and columns clustering.

### 4.6. Motif Enrichment Analysis

To perform motif enrichment analysis at the first step we extracted 3′UTR sequences of genes differentially expressed after NANOS1 and/or NANOS3 overexpression. To minimize different isoforms bias we selected the highest expressed transcript, using salmon version 0.12.0 [69] for quantification in control (empty vector transfected) cells. Salmon index was created with the following command: salmon index -t/path/to/transcriptome/gencode.v26.transcripts_simple.fa.gz -i gencodev26 -k 31. Salmon was used with the following parameters: salmon quant -l IU -i/path/to/index -1 trimmed_read1.fq -2 trimmed_read2.fq --validateMappings -o/path/to/output. Results from three replicates were merged with salmon quantmerge and isoform with the highest mean TPM for each gene was chosen. Then, 3′UTR sequences were extracted using threeUTRsByTranscript function from GenomicFeatures R library version 1.38.2 [70] with GRCh38 genome annotation from gencode version 26, and written as fasta files. All T’s in 3′UTR sequences were replaced by U’s with sed -i ‘s/T/U/g’ < input_3UTR_seqs.fa command. Motif enrichment analysis was performed using STREME version 5.4.0 [71] with the following parameters: streme --p/path/to/input_3UTR_seqs.fa --order 1 --rna --minw 4 --maxw 10 --thresh 0.05 --oc/path/to/output (dinucleotide-shuffled sequences were used as a background, minimum motif width = 4, maximum motif width = 10, *p* value threshold = 0.05).

### 4.7. Transcription Factor Prediction

Transcription factor prediction was performed in Cytoscape v3.6.1 using iRegulon v1.3 [40] using default parameters.

### 4.8. Western Blot

SDS lysates were resolved in 10% SDS polyacrylamide gels and transferred to nitrocellulose membranes (BioRad (Hercules, CA, USA) #1620112). Membranes were blocked with 5% low-fat milk in the TBS buffer supplemented with 0.1% Tween 20 (blocking buffer) at RT for 1 h. Membranes were incubated with primary antibodies at 4 °C overnight in the blocking buffer. After blocking, membranes were washed 4 times in TBS buffer with 0.1% Tween 20 and incubated with horseradish peroxidase (HRP)-conjugated secondary antibodies at RT for 1 h in the same buffer. Next, membranes were washed twice in TBS buffer with 0.1% Tween 20, and then twice in TBS buffer. Clarity™ ECL Western Blotting Substrate (BioRad #1705060) and the ChemiDoc Touch Imaging System (BioRad) were used for signal development. List of the antibodies used and their concentrations are presented in Table S8.

### 4.9. Cell Cycle Assay

Cell cycle analysis was performed 24 h following NANOS1 and NANOS3 overexpression with the corresponding negative controls in 3 biological replicates. TCam-2 cells were washed with PBS and fixed in cold 100% methanol overnight in −20 °C. Following this step, cells were incubated with 0.1 µg/mL propidium iodide (PI) (Sigma Aldrich, St. Louis, MO, USA, #P4170) and 330 µg/mL RNase A (Sigma Aldrich #R6513) at 37 °C for 15 min followed by another incubation on ice for 1 h and were analyzed using the S3e™ Cell Sorter (BioRad) equipment. Output data were analyzed using ModFit LT (Verity Software House).

### 4.10. Apoptosis Assay

Detection of apoptotic TCam-2 cells was performed 48 h following NANOS1 and NANOS3 overexpression with corresponding negative controls in two biological replicates using the Annexin V-FITC Apoptosis Detection Kit (Beckman Coulter, Indianapolis, IN, USA, #IM3546) according to the manufacturer’s protocol, followed by flow cytometry using the FlowSight apparatus (Amnis, Seattle, WA, USA). Results were analyzed using Image Data Exploration and Analysis Software (IDEAS^®^; Amnis, Seattle, USA).

### 4.11. Luciferase Reporter Assay

Luciferase reporter assays are performed as described previously [25]. Briefly, HEK293FT cells were co-transfected with psiCheck2 vectors (Promega, Madison, WI, USA, #C802A) and with plasmids/siRNA, in 1:10 ratio to induce overexpression of NANOS3 and knockdown of PUM1 in three biological replicates. After 48 h the cells were lysed, and luciferase luminescence was measured using the Glomax-Multi Detection System and Dual-luciferase Reporter Kit (Promega #E1980) according to the manufacturer’s protocol. The mean ratio of Renilla to Firefly luminescence for each sample is presented as a percentage of the relative luciferase units (RLU). Standard errors were shown as error bars. Two-tailed *t* test was used to determine statistical significance.

### 4.12. GST Pull-Down

Upon overexpression of NANOS3-GST, TCam-2 cells were washed with PBS, followed by 0.3% formaldehyde (Thermo Fisher Scientific #252549) crosslinking for 10 min. Crosslinking reaction was stopped by using glycine solution for 15 min on ice. Cells were lysed for 30 min on ice with 1 mL of 1% Triton X-100 buffer supplemented with protease inhibitors. Then, 100 µ/mL of RNase A or 1x RNase inhibitors were added to full lysis for RNase + and − samples respectively. Lysates were centrifuged at 15,000× *g* at 4 °C for 15 min, followed by 2 h incubation (at 4 °C with rotation) of the supernatant with Pierce GST beads (Thermo Fisher Scientific #78601). Beads were washed three times with TBS buffer, followed by another three washes with the lysis buffer and resuspended in SDS-PAGE sample buffer for Western blot analysis.

### 4.13. ChIP-qPCR

Chromatin immunoprecipitation followed by qPCR was performed as described previously [72]. Briefly, TCam-2 cells in 2 biological replicates were fixed with 1% formaldehyde for 15 min and crosslinking was stopped by adding 0.125 M glycine solution. Further steps were performed by Active Motif. In total, 40 µg of chromatin and 25 µL of FOXM1 antibody (Santa Cruz, cat # sc-502) were used for each ChIP reaction. List of the primers used are presented in Table S9.

### 4.14. Differential Correlation Analysis

Healthy testis and testis cancer expression datasets were obtained from GTEx (*n* = 361) and TCGA (*n* = 157) databases respectively. GTEx and TCGA use different gene expression units in their datasets—TPM and FPKM, respectively. Therefore, TCGA expression data were transformed from FPKM to TPM using the following equation: TPM = gene of interest FPKM/sum (all genes FPKM) × 10^6^. Differential correlation analysis was performed using DGCA package [73] in R version 3.6.3.

## 5. Conclusions

Our study provides the first transcriptomic characterization upon induced expression of NANOS1 and NANOS3 in a human germ cell model with an emphasis on mRNAs related to infertility and cancer, many of which carry out functions related to the cell cycle. Moreover, we show that NANOS1 and NANOS3 have distinct effects on the cell cycle. Lastly, we demonstrate the role of the NANOS3–PUM1–FOXM1 axis in regulating mRNAs involved in G2/M phase and propose dysregulation of this axis as a factor contributing to testis cancers.

## Data Availability

RNA-Seq data described in this study are available from the Gene Expression Omnibus (accession GSE134802 and GSE123016). The rest of the data and R code used for analysis are available upon request from the corresponding authors.

## Supplementary Materials

The following supporting information can be downloaded at: https://www.mdpi.com/article/10.3390/ijms23126592/s1.
