# Supplementary material for: Distinct Roles of NANOS1 and NANOS3 in the Cell Cycle and NANOS3-PUM1-FOXM1 Axis to Control G2/M Phase in a Human Primordial Germ Cell Model
Source: Int J Mol Sci. 2022 Jun 13;23(12):6592. doi: 10.3390/ijms23126592 (PMC9223905; doi:10.3390/ijms23126592)
Supplement: Supplementary file 1 [file ijms-23-06592-s001.zip › Supplementary Figures.pdf]

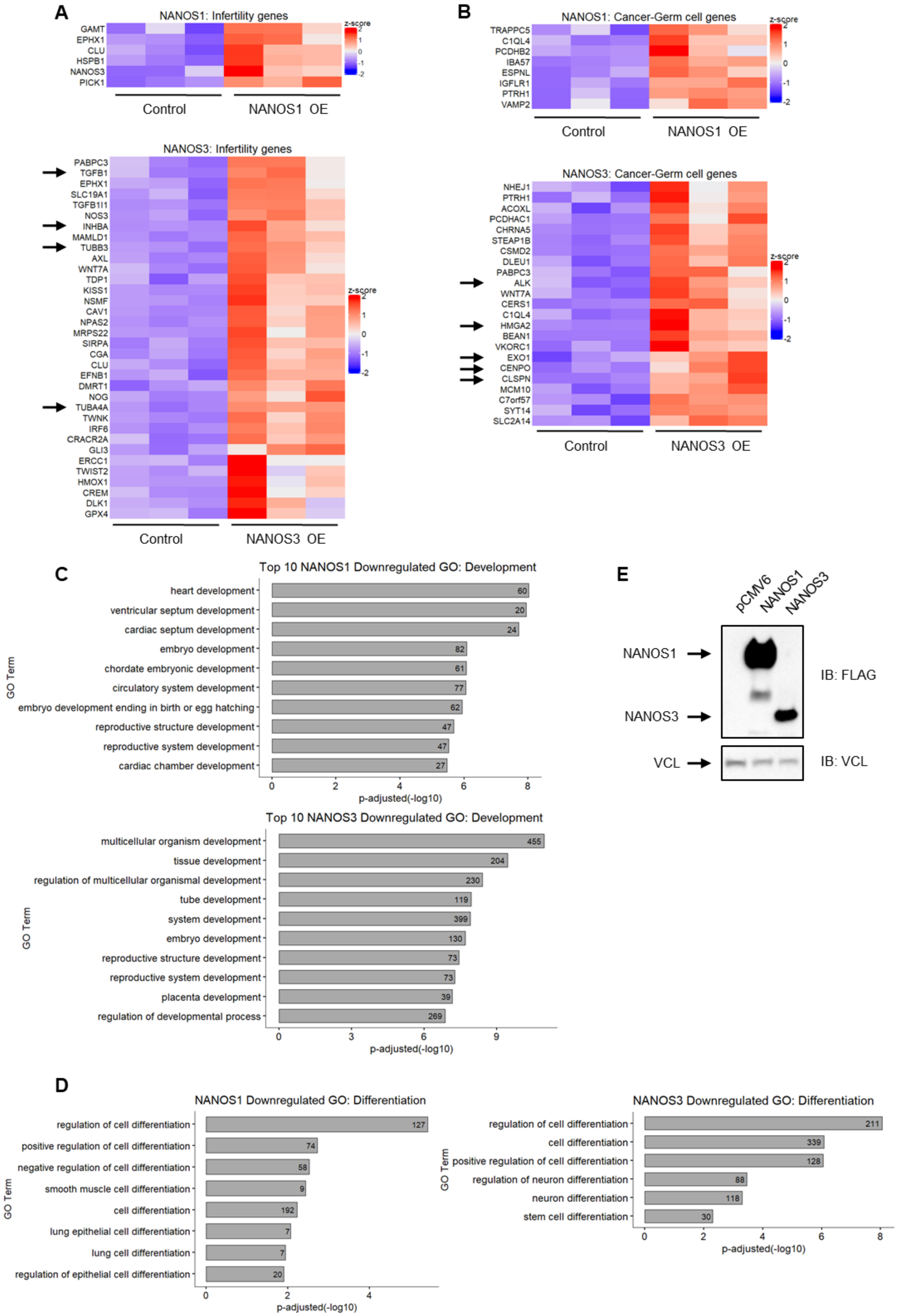
A
NANOS1: Infertility genes
GAMT
EPHX1
CLU
HSPB1
NANOS3
PICK1
z-score
2
1
0
-1
-2
Control
NANOS1 OE
NANOS3: Infertility genes
PABPC3
TGFB1
EPHX1
SLC19A1
TGFB1I1
NOS3
INHBA
MAMLD1
TUBB3
AXL
WNT7A
TDP1
KISS1
NSMF
CAV1
NPAS2
MRPS22
SIRPA
CGA
CLU
EFNB1
DMRT1
NOG
TUBA4A
TWNK
IRF6
CRACR2A
GLI3
ERCC1
TWIST2
HMOX1
CREM
DLK1
GPX4
z-score
Control
NANOS3 OE
B
NANOS1: Cancer-Germ cell genes
TRAPPC5
C1QL4
PCDHB2
IBA57
ESPNL
IGFLR1
PTRH1
VAMP2
z-score
Control
NANOS1 OE
NANOS3: Cancer-Germ cell genes
NHEJ1
PTRH1
ACOXL
PCDHAC1
CHRNA5
STEAP1B
CSMD2
DLEU1
PABPC3
ALK
WNT7A
CERS1
C1QL4
HMGA2
BEAN1
VKORC1
EXO1
CENPO
CLSPN
MCM10
C7orf57
SYT14
SLC2A14
z-score
Control
NANOS3 OE
C
Top 10 NANOS1 Downregulated GO: Development
heart development 60
ventricular septum development 20
cardiac septum development 24
embryo development 82
chordate embryonic development 61
circulatory system development 77
embryo development ending in birth or egg hatching 62
reproductive structure development 47
reproductive system development 47
cardiac chamber development 27
GO Term
p-adjusted(-log10)
0 2 4 6 8
Top 10 NANOS3 Downregulated GO: Development
multicellular organism development 455
tissue development 204
regulation of multicellular organismal development 230
tube development 119
system development 399
embryo development 130
reproductive structure development 73
reproductive system development 73
placenta development 39
regulation of developmental process 269
GO Term
p-adjusted(-log10)
0 3 6 9
D
NANOS1 Downregulated GO: Differentiation
regulation of cell differentiation 127
positive regulation of cell differentiation 74
negative regulation of cell differentiation 58
smooth muscle cell differentiation 9
cell differentiation 192
lung epithelial cell differentiation 7
lung cell differentiation 7
regulation of epithelial cell differentiation 20
GO Term
p-adjusted(-log10)
0 2 4
NANOS3 Downregulated GO: Differentiation
regulation of cell differentiation 211
cell differentiation 339
positive regulation of cell differentiation 128
regulation of neuron differentiation 88
neuron differentiation 118
stem cell differentiation 30
GO Term
p-adjusted(-log10)
0 2 4 6 8
E
pCMV6
NANOS1
NANOS3
NANOS1
NANOS3
IB: FLAG
VCL
IB: VCL

Supplementary Figure S1. Transcriptomic changes upon NANOS1 and NANOS3 overexpression**. A)** Infertility genes upregulated upon NANOS1 (upper heatmap) and NANOS3 (lower heatmap) overexpression. Genes involved in cell cycle are marked by arrows. **B)** Cancer-germ cell genes upregulated upon NANOS1 (upper heatmap) and NANOS3 (lower heatmap) overexpression. Genes involved in cell cycle are marked by arrows. **C)** Top 10 development related biological processes identified by gene ontology (GO) analysis upon NANOS1 (top panel) and NANOS3 (bottom panel) overexpression. **D)** Top 10 differentiation related biological pro-cesses identified by gene ontology (GO) analysis upon NANOS1 (top panel) and NANOS3 (bottom panel) overexpression. **E)** Representative western blot of NANOS1 and NANOS3 overexpression for RNA-Sequencing analysis.

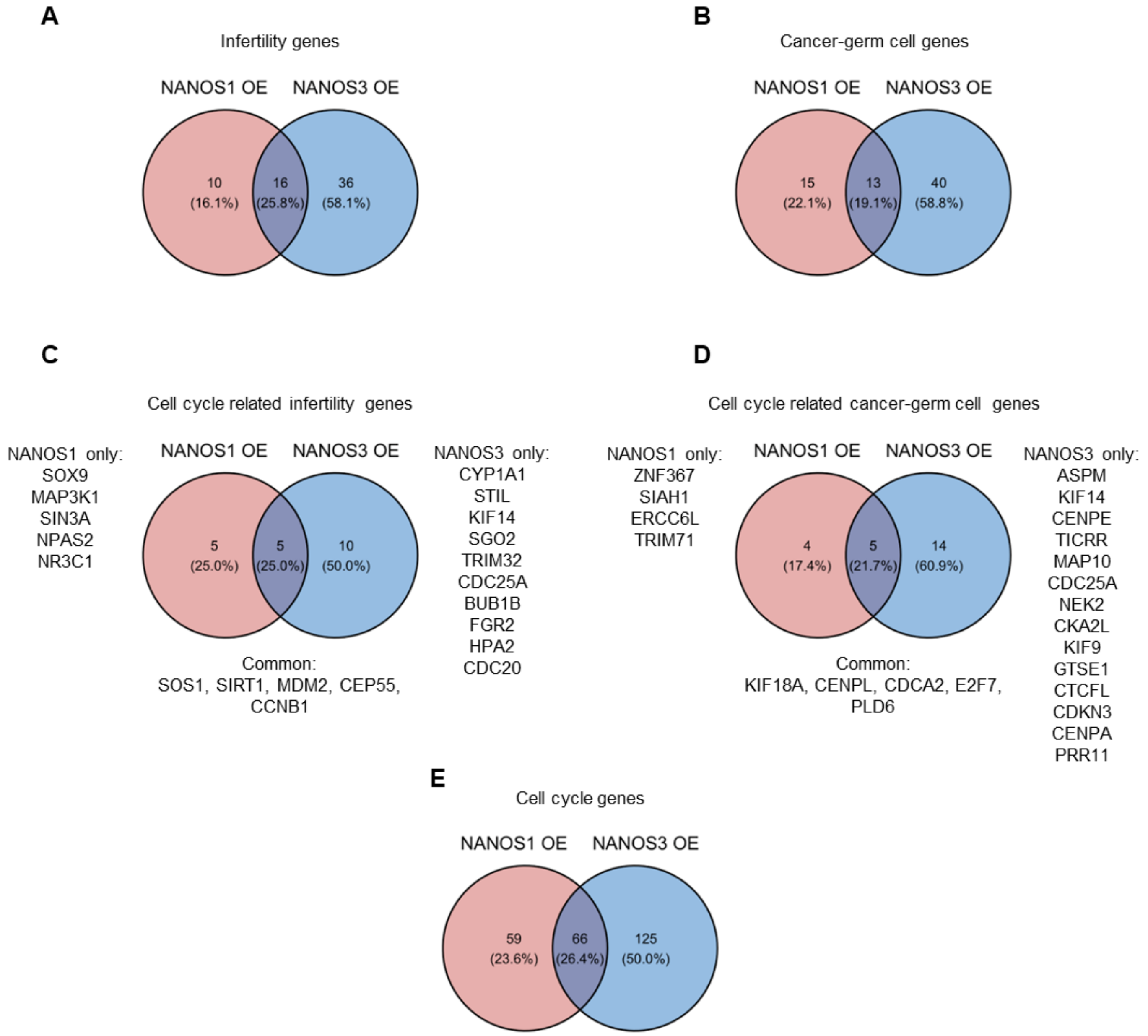

Supplementary Figure S2. Venn diagrams showing common and distinct downregulated **A)** infertility, **B)** cancer-germ cell, **C)** cell cycle related infertility, **D)** cell cycle related cancer-germ cell and **E)** cell cycle genes.

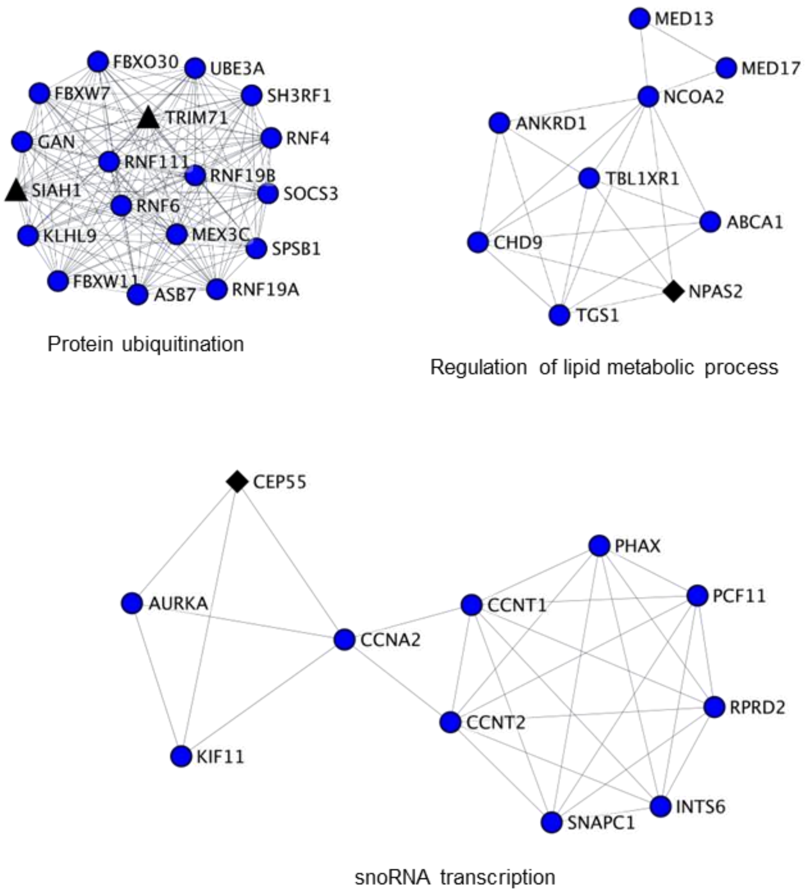

Supplementary Figure S3. NANOS1 downregulated BCA clusters containing infertility (black diamonds) and cancer-germ cell genes (black triangles).

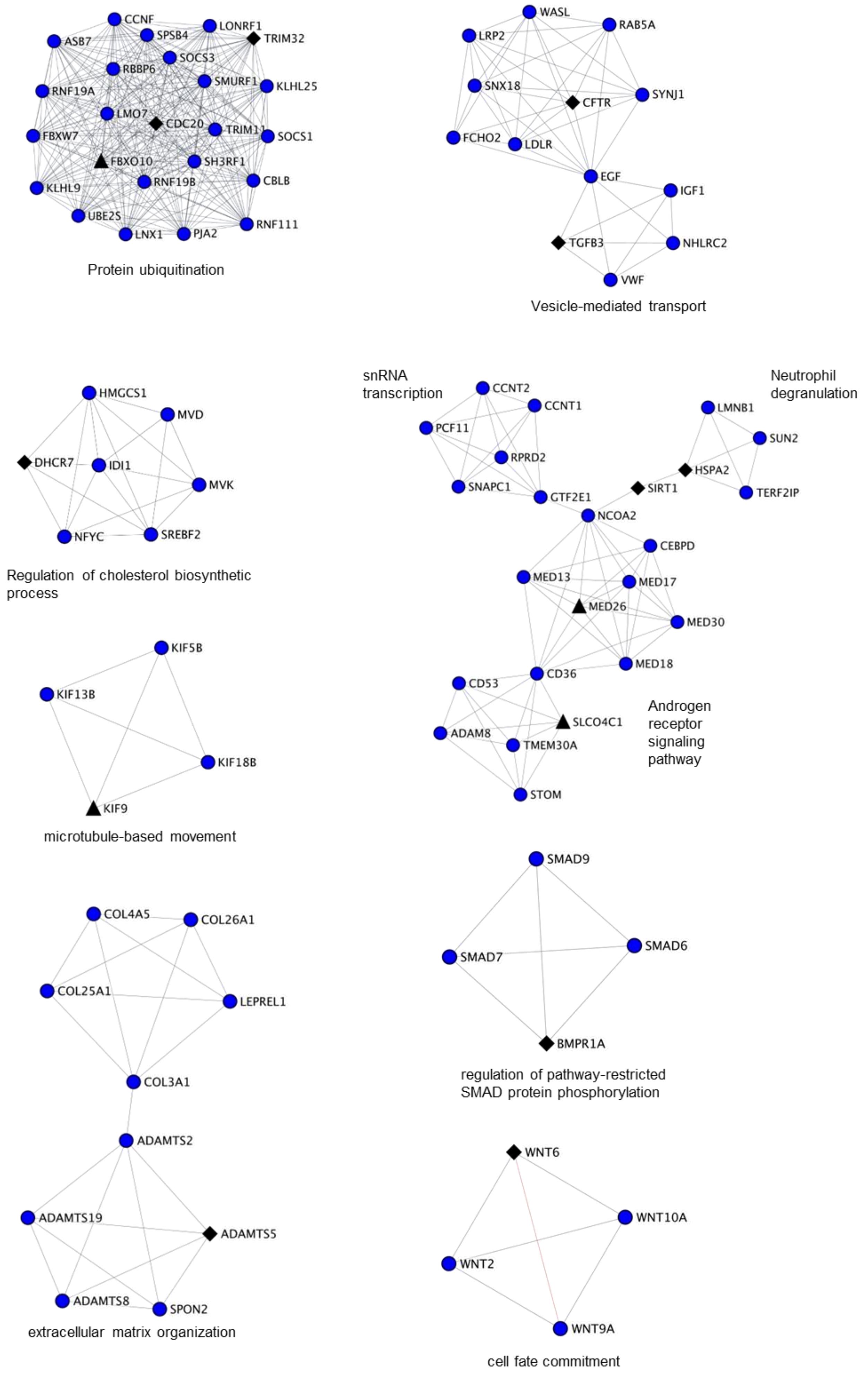
CCNF
LONRF1
ASB7
SPSB4
TRIM32
SOCS3
RBBP6
SMURF1
RNF19A
KLHL25
LMO7
CDC20
TRIM11
FBXW7
SOCS1
FBXO10
SH3RF1
RNF19B
CBLB
KLHL9
UBE2S
RNF111
LNX1
PJA2
Protein ubiquitination
WASL
LRP2
RAB5A
SNX18
SYNJ1
CFTR
FCHO2
LDLR
EGF
IGF1
TGFB3
NHLRC2
VWF
Vesicle-mediated transport
HMGCS1
MVD
DHCR7
IDI1
MVK
NFYC
SREBF2
Regulation of cholesterol biosynthetic process
snRNA transcription
CCNT2
CCNT1
PCF11
RPRD2
SNAPC1
GTF2E1
Neutrophil degranulation
LMNB1
SUN2
HSPA2
SIRT1
TERF2IP
NCOA2
CEBPD
MED13
MED17
MED26
MED30
MED18
CD36
CD53
SLCO4C1
ADAM8
TMEM30A
STOM
Androgen receptor signaling pathway
KIF5B
KIF13B
KIF18B
KIF9
microtubule-based movement
SMAD9
SMAD7
SMAD6
BMPR1A
regulation of pathway-restricted SMAD protein phosphorylation
COL4A5
COL26A1
COL25A1
LEPREL1
COL3A1
ADAMTS2
ADAMTS19
ADAMTS5
ADAMTS8
SPON2
extracellular matrix organization
WNT6
WNT10A
WNT2
WNT9A
cell fate commitment

Supplementary Figure S4. NANOS3 downregulated BCA clusters containing infertility (black diamonds) and cancer-germ cell genes (black triangles).

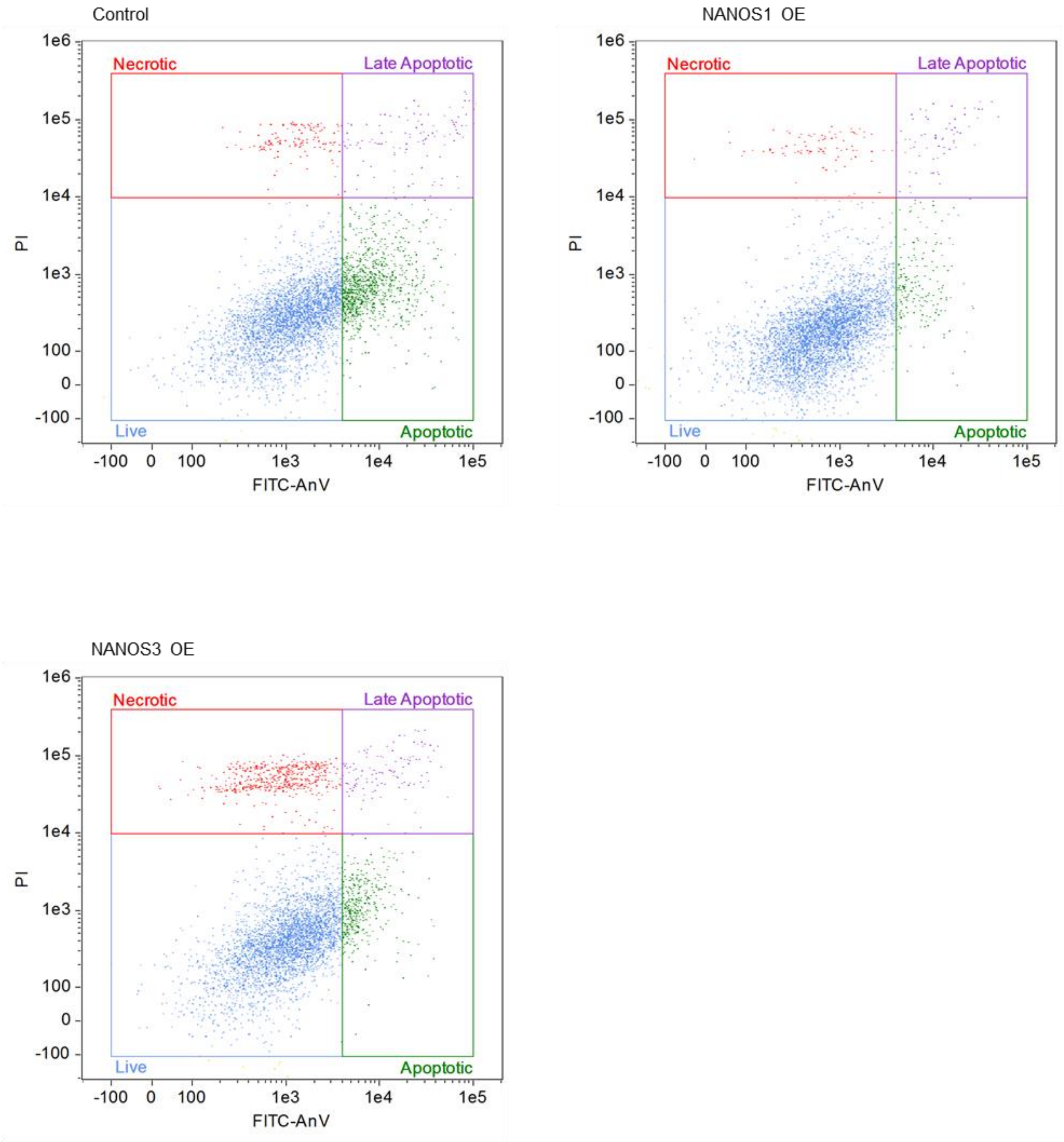

Supplementary Figure S5. Flow-cytometry representative scatterplots of Annexin V apoptosis analysis.

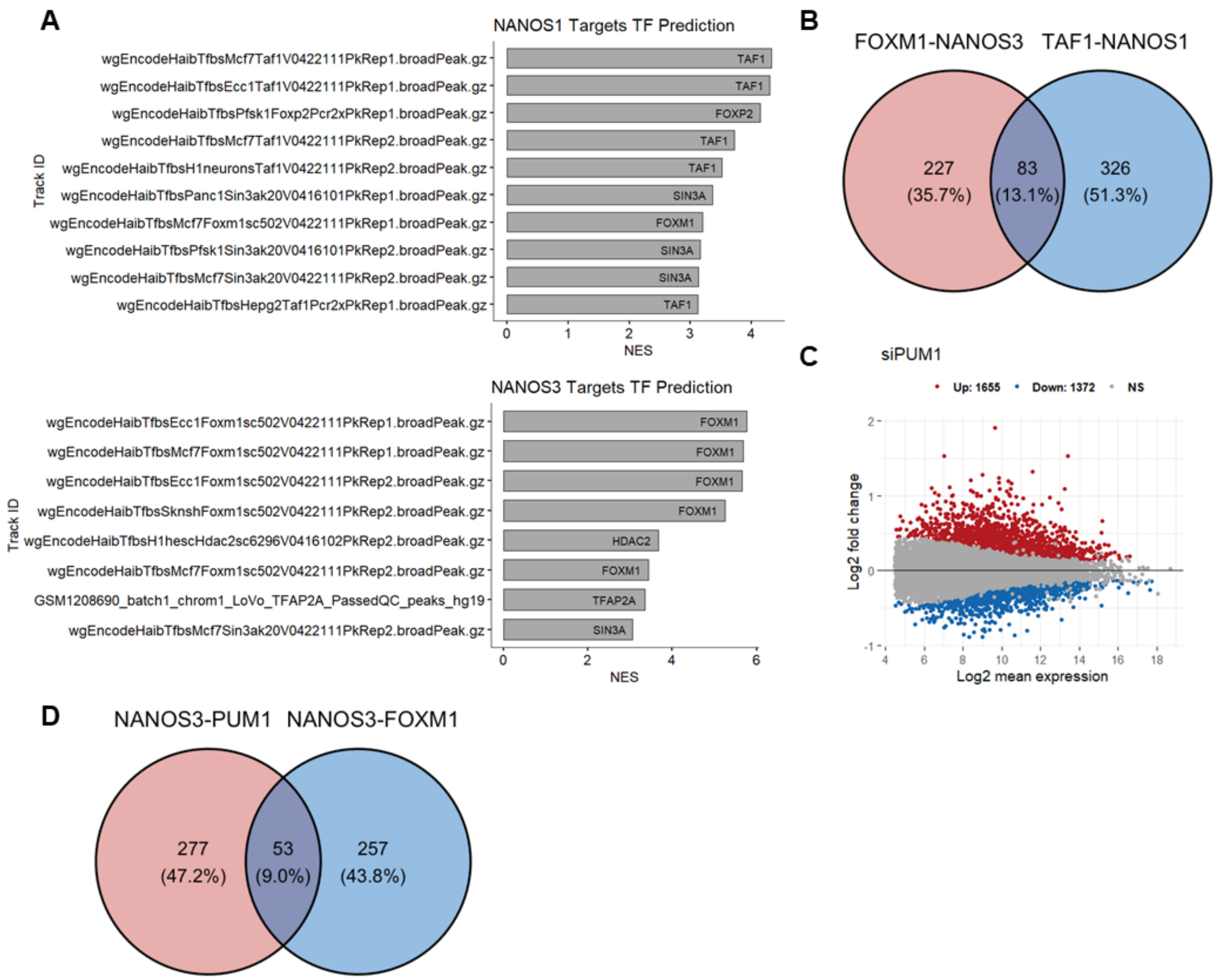

Supplementary Figure S6. FOXM1 and TAF1 act as transcription factors for NANOS1 and NANOS3 downregulated genes. **A)** iRegulon transcription factor prediction analysis on NANOS1 downregulated (top panel), NANOS3 downregulated (bottom panel) genes ranked by the normalized enrichment score (NES). **B)** Venn diagram showing the distinct and common genes idenfitied by iRegulon to be regulated by FOXM1-NANOS3 and TAF1-NANOS1 pairs. **C)** RNA-Sequencing (RNA-Seq) upon PUM1 knock-down visualized as an MA-plot. Differentially expressed genes were filtered with log2FC ≥ 0.5 for upregulated and ≤ -0.5 for downregulated genes. Adjusted p-value ≤ 0.01 was considered as significant. **D)** Venn diagram showing distinct and common genes regulated by NANOS3-PUM1 and NANOS3-FOXM1 pairs.

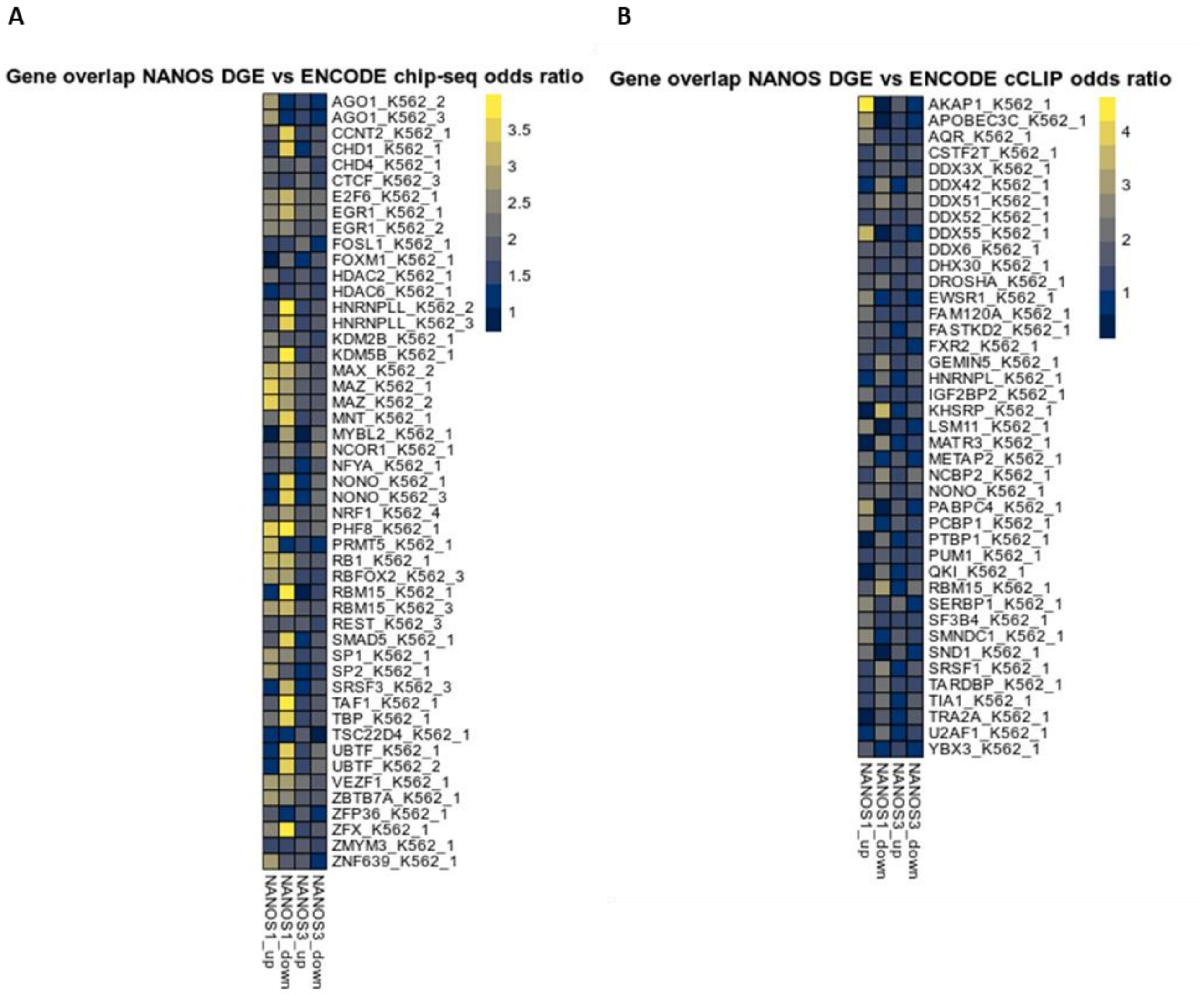

Supplementary Figure S7. Gene overlap results of ENCODE **A)** ChIP-Seq and **B)** eCLIP data from K562 cell line. Gene overlaps with p value < 0.01 were considered as significant. Top 15 significant overlaps with the highest odds ratio for each category were visualized.

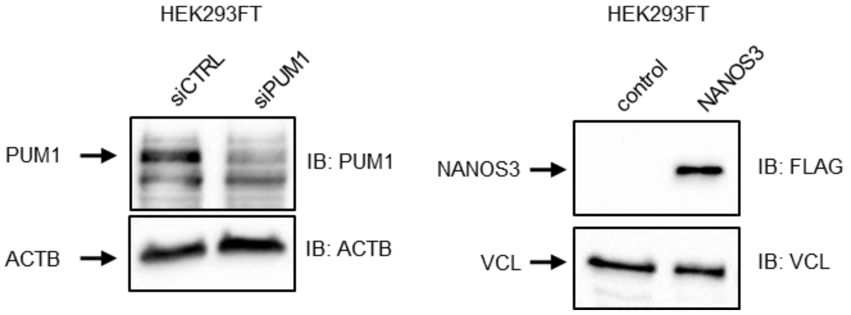

Supplementary Figure S8. Representative western blot results showing PUM1 silencing and NANOS3 overexpression performed for dual luciferase experiments.
